# cEEG and rEEG detection rates of prognostic indicators in cardiac arrest patients: a systematic review and diagnostic meta-analysis

**DOI:** 10.3389/fneur.2026.1760363

**Published:** 2026-02-18

**Authors:** Jiayi Lin, Zhonghao Ji, Xue Lin, Yihan Yan, Tianbo Zheng, Hanyang Zhang, Yiting Wu, Yuru Wang, Zihan Yu, Haibo Di, Nantu Hu

**Affiliations:** 1 Zhejiang-Belgium Joint Laboratory for Disorders of Consciousness, Hangzhou, China; 2 International Unresponsive Wakefulness Syndrome and Consciousness Science Institute, Hangzhou Normal University, Hangzhou, China; 3 School of Clinical Medicine, Hangzhou Normal University, Hangzhou, China

**Keywords:** cardiac arrest, EEG, electroencephalography, meta-analysis, prognostic

## Abstract

**Introduction:**

Accurate neuroprognostication following cardiac arrest is essential for clinical decision-making; however, the comparative diagnostic performance of continuous electroencephalography (cEEG) and routine electroencephalography (rEEG) remains uncertain. Although cEEG is preferred for the detection of dynamic electrographic abnormalities such as nonconvulsive status epilepticus, the implementation of this technique is limited by high resource demands. Whether rEEG, a typically brief (20–30 min) recording, provides comparable prognostic accuracy is still debated.

**Methods:**

We searched PubMed, Embase, Web of Science, and the Cochrane Library from January 2010 to December 2024 for studies relating to comatose post-cardiac arrest patients. Methodological quality was assessed using quality assessment of diagnostic accuracy studies 2 (QUADAS-2) and QUADAS-C. Statistical analyses were performed using Stata v18.0, with pooled sensitivity, specificity, and area under the summary receiver operating characteristic (SROC) curve (AUC) was estimated using random-effects models.

**Results:**

Sixteen studies (5,895 patients) were included. cEEG exhibited a pooled sensitivity of 0.53 [95% confidence interval (CI): 0.45–0.61] and specificity of 0.99 (95% CI: 0.97–1.00; AUC = 0.85). rEEG yielded a sensitivity of 0.50 (95% CI: 0.42–0.58) and a specificity of 0.97 (95% CI: 0.92–0.99; AUC = 0.75). Sensitivity analyses confirmed robustness while Deeks’ test indicated low publication bias (cEEG: *p* = 0.48; rEEG: *p* = 0.05).

**Discussion:**

Despite the theoretical advantages of cEEG in monitoring evolving brain activity, rEEG demonstrated comparable diagnostic performance, particularly in specificity, with substantially lower resource requirements. Our findings suggest that rEEG may serve as a feasible alternative or complementary tool to cEEG, especially in resource-constrained or time-sensitive settings, thereby supporting more accessible EEG-based neuroprognostication.

**Systematic review registration:**

https://www.crd.york.ac.uk/PROSPERO/view/CRD420251151755, CRD420251151755.

## 1 Introduction

Cardiac arrest (CA) is one of the leading cardiovascular emergencies worldwide, resulting in up to 20% of global deaths (1, 2). The pathogenesis of CA is complex and is characterized primarily by systemic multi-organ ischemia–reperfusion injury, which frequently culminates in organ dysfunction or failure (3). Over recent years, continual refinements in the concepts of cardiopulmonary resuscitation and the optimization of advanced life support systems have markedly increased the rate of return of spontaneous circulation (ROSC) to approximately 37% (95% confidence interval: 23–54%) (4). Nevertheless, despite improved survival, 45–70% of resuscitated patients develop hypoxic–ischemic brain injury, and approximately 30% of patients ultimately sustain severe neurological impairment, reflected by Cerebral Performance Category (CPC) scores of 3–5 (5, 6). This type of brain injury leads to disorders of consciousness and cognition and, given the high medical costs and intensive caregiving requirements, constitutes a central challenge in neurocritical care.

During prognostic assessment after CA, a number of variables, including age, initial cardiac rhythm (shockable *versus* non-shockable), and secondary seizures, including nonconvulsive status epilepticus (NCSE), can influence neurological outcomes *via* distinct mechanisms. Recent evidence demonstrated that among patients with ventricular fibrillation (VF), one-month survival and the proportion of patients achieving a CPC 1–2 were 23 and 17%, respectively, whereas the corresponding figures for patients with pulseless electrical activity (PEA) were 9 and 3% (7). These data indicated that VF is associated with a better neurological prognosis than PEA.

No single clinical indicator can fully capture the dynamic evolution of brain injury. Accordingly, international guidelines, such as those of the American Heart Association and the European Resuscitation Council, strongly recommend multimodal assessment integrating clinical features, neurophysiological data, molecular biomarkers, and neuroimaging (8). This multimodal approach improves prognostication after CA, particularly with respect to neurological recovery. By providing continuous and real-time information on cortical activity, electroencephalography (EEG) provides unique advantages for evaluating the severity of brain injury and informing prognosis (9), and thus is crucial in multimodal evaluation frameworks.

First, EEG has significant value for early-warning value and can identify early abnormalities such as an unreactive background, status epilepticus, and low-voltage patterns, which are closely associated with poor outcomes (10). Previous studies have shown that early EEG monitoring within 24 h exhibits high sensitivity and specificity for predicting neurological outcomes after CA (11). Moreover, the absence of EEG reactivity is regarded as an independent predictor of an unfavorable prognosis (12). Second, compared with clinical examinations that are subject to observer variability, quantitative techniques, such as spectral analysis, provide objective and quantifiable EEG parameters, such as high-frequency spikes and connectivity alterations that more accurately reflect brain function (13). Third, EEG is flexible and can be used alone or combined with clinical indicators (e.g., pupillary reflexes, pain responses), imaging (e.g., computed tomography, magnetic resonance imaging), and biomarkers [e.g., neuron-specific enolase (NSE)] to construct multimodal predictive models (14) that substantially enhance accuracy. Finally, standardized EEG terminology developed by the American Clinical Neurophysiology Society (ACNS) provides consistent evaluative criteria across studies (15), thus improving the practicality and comparability of EEG in multimodal prognostication. Collectively, these features reinforce EEG as an indispensable tool for neuroprognostic assessment following CA.

Over recent years, research has increasingly focused on comparing rEEG (16), typically a single 20–30-min recording, with cEEG (17), which generally lasts 24–48 h or even several days. Relative to rEEG, recent evidence has shown that cEEG provides higher sensitivity for detecting seizures (particularly NCSE) and for characterizing background EEG patterns; accordingly, the ACNS recommends cEEG monitoring for CA patients (18).

Despite these advantages, cEEG faces several limitations in clinical practice. First, resource consumption and cost are substantially higher than for rEEG, including sustained monitoring equipment, specialized personnel, and greater hospital resources; these factors particularly limit the adoption of this strategy in resource-constrained settings. Second, although cEEG provides more detailed EEG information, the incremental clinical value of this technique has yet to be fully validated and previous studies have not consistently demonstrated that cEEG improves patient outcomes. Therefore, existing evidence is insufficient to support the universal promotion of cEEG across all clinical scenarios (19).

In contrast, rEEG demonstrates unique practical value. For patients who cannot tolerate prolonged monitoring, repeated rEEG recordings can efficiently obtain key prognostic information (20). Existing studies further suggest that rEEG performs comparably to cEEG in terms of prognostic discrimination (21, 22), with superior cost-effectiveness in resource-limited or cost-sensitive contexts (23, 24). These findings provide a robust clinical rationale for optimizing EEG monitoring strategies to achieve precise yet economical patient management.

The current controversy over whether cEEG is superior to rEEG centers on whether intensifying monitoring improves prognostic assessment in a meaningful manner. Due to the lack of large, rigorously designed, multicenter, randomized and controlled trials, conclusive evidence that cEEG outperforms rEEG for prognostication is still lacking. Therefore, in the present study, we used meta-analytic methods to comprehensively compare the diagnostic value of rEEG *versus* cEEG for predicting outcomes after CA. Our findings are expected to guide the clinical selection of optimal EEG monitoring strategies under varying conditions, balancing diagnostic accuracy with rational resource allocation.

## 2 Materials and methods

This systematic review was conducted in accordance with the Preferred Reporting Items for Systematic Reviews and Meta-Analyses (PRISMA) guidelines (25) for diagnostic test accuracy reviews (Supplementary material) and was prospectively registered in PROSPERO (Reference: CRD420251151755).

### 2.1 Inclusion criteria

#### 2.1.1 Patient population

The inclusion criteria were as follows: (1) patients admitted with coma following CA; patients with unresponsive wakefulness syndrome or a Glasgow Coma Scale (GCS) score ≤8 were considered comatose; and (2) studies including patients with hypoxic coma due to causes other than CA.

The exclusion criteria were as follows: (1) individuals younger than 18 years; (2) patients with a previous history of epilepsy or related disorders; and (3) failure to achieve sustained ROSC after admission.

#### 2.1.2 Interventions

Differences in standard in-hospital care after admission, such as targeted temperature management, were not considered in this review. Eligible cohorts were those in which EEG assessment (primarily cEEG and rEEG) was performed.

#### 2.1.3 Outcomes

The primary outcome was the neurological outcome rated at specified time points after admission using the CPC scale; CPC 1–2 was defined as a favorable outcome, and CPC 3–5 as an unfavorable outcome (26).

The secondary outcomes included mortality, quality-of-life, and adverse events.

#### 2.1.4 Study designs

Eligible designs included randomized controlled trials, prospective nonrandomized studies, observational studies, cohort studies, and retrospective studies that met the above inclusion criteria. Meta-analyses, systematic reviews, narrative reviews, pathology reports, and conference abstracts were excluded because methodological quality could not be adequately assessed.

#### 2.1.5 Scope of EEG assessment

The meta-analysis presented focuses on comparing the overall diagnostic performance of two EEG monitoring modalities (cEEG vs. rEEG) for predicting unfavorable outcomes after cardiac arrest. However, this study does not aim to evaluate the sensitivity, specificity, or prognostic value of individual EEG patterns separately. Individual EEG patterns include, but are not limited to, unreactive background, NCSE, low-voltage patterns, and spectral abnormalities. We synthesized the diagnostic accuracy of each EEG modality, instead of analyzing specific EEG patterns. The scope of the study aligns with the core objectives, providing evidence for the rational selection of EEG monitoring strategies in clinical practice.

### 2.2 Search strategy and selection criteria

Following the PICO framework (participants, interventions, comparisons and outcomes) (27) and with reference to other systematic reviews comparing the cEEG and rEEG detection rates of prognostic indicators in CA, we developed a comprehensive search strategy. We searched PubMed, Embase, Web of Science, and the Cochrane Library for literature published from January 1 2010 to December 31, 2024. The strategy was developed collaboratively by LJY, LX, and YYH and reviewed by an experienced neurologist (HNT) to enhance search quality. Population terms (“Heart arrest,” “Hypoxia-Ischemia, Brain,” or “Coma”) were combined with intervention terms (“cEEG” or “rEEG”). Specific details of the search strategy are provided in Supplementary material. Initial records were imported into EndNote v20.0 (Clarivate, Philadelphia, PA, United States). Two independent authors (LJY and YYH) screened titles and abstracts against eligibility criteria to identify potentially relevant studies, followed by independent full-text review of jointly selected records to minimize bias. Discrepancies were adjudicated by a third reviewer (LX).

### 2.3 Data extraction and quality assessment

Two independent reviewers (LJY and JZH) extracted data into a standardized MS Excel form (Microsoft Corporation, Redmond, WA, United States), including authorship, publication year, study design, population characteristics, and diagnostic contingency data [true positive (TP), false positive (FP), false negative (FN), and true negative (TN)]. Eligible trials were appraised using the QUADAS-2 checklist (Supplementary material) and its comparative extension (QUADAS-C) across four domains: patient selection, index test, reference standard, and flow/timing (28, 29). Risk of bias (RoB) was judged for each domain, and applicability concerns were evaluated for the first three domains. For patient selection, studies that did not specify consecutive or random recruitment were considered to be associated with a high RoB. For the index test domain, if EEG readers analyzing cEEG or rEEG patterns were not blinded to the patient’s clinical context, other neuroprognostic test results, or final outcomes, interpretive bias was considered present and such studies were rated as been associated with a high RoB. For the reference standard, because all included studies used commonly accepted prognostic tools [e.g., CPC, modified Rankin Scale (mRS), or neurological examination] as primary endpoints, and because these assessments were independent of EEG interpretation without the incorporation of index-test results, the RoB for this domain was deemed low for all studies. With regards to flow and timing, studies that did not clearly report the timing of EEG relative to CA occurrence, the completion of resuscitation, or the completion of temperature management were considered to provide incomplete information relating to patient flow and scheduling and were rated as having a high RoB in this domain. When insufficient information precluded the complete evaluation of any domain, RoB was judged as unclear. All papers were assessed independently by two authors (LJY and JZH), with disagreements resolved by a third author (LX).

### 2.4 Statistical methods

We performed meta-analyses using Stata v18 (StataCorp, College Station, TX, United States). For each study included in our final analysis, we estimated pooled sensitivity, specificity, diagnostic odds ratio (DOR) with 95% confidence intervals (CIs), and the area under the SROC curve (AUC) to evaluate diagnostic accuracy. Pooled estimates are reported with 95% CIs and all tests were two-sided with statistical significance set at *p* < 0.05. Between-study heterogeneity was assessed using the *I*^2^ statistic (30). AUC values were interpreted as follows: 0.5, non-informative test; 0.5–0.7, low accuracy; 0.7–0.9, moderate accuracy; 0.9–1.0, high accuracy; and 1.0, perfect test (31). Given substantial heterogeneity, random-effects models were applied for sensitivity analyses. Publication bias was assessed using Deeks’ test.

### 2.5 Handling of multiple data points from a single study

For studies reporting predictive performance at multiple time points after CA, we treated each EEG assessment as an independent data point in the quantitative synthesis. This decision was based on the clinical and biological premise that recordings obtained at different time windows capture distinct pathophysiological stages of post–cardiac arrest brain injury and are interpreted within different clinical decision-making contexts; thus, they effectively address related but non-identical prognostic questions. Accordingly, these time-specific estimates were included separately and pooled using random-effects models. The robustness of the resulting summary estimates was further evaluated through sensitivity analyses.

## 3 Results

### 3.1 Literature search results

**Figure 1:**
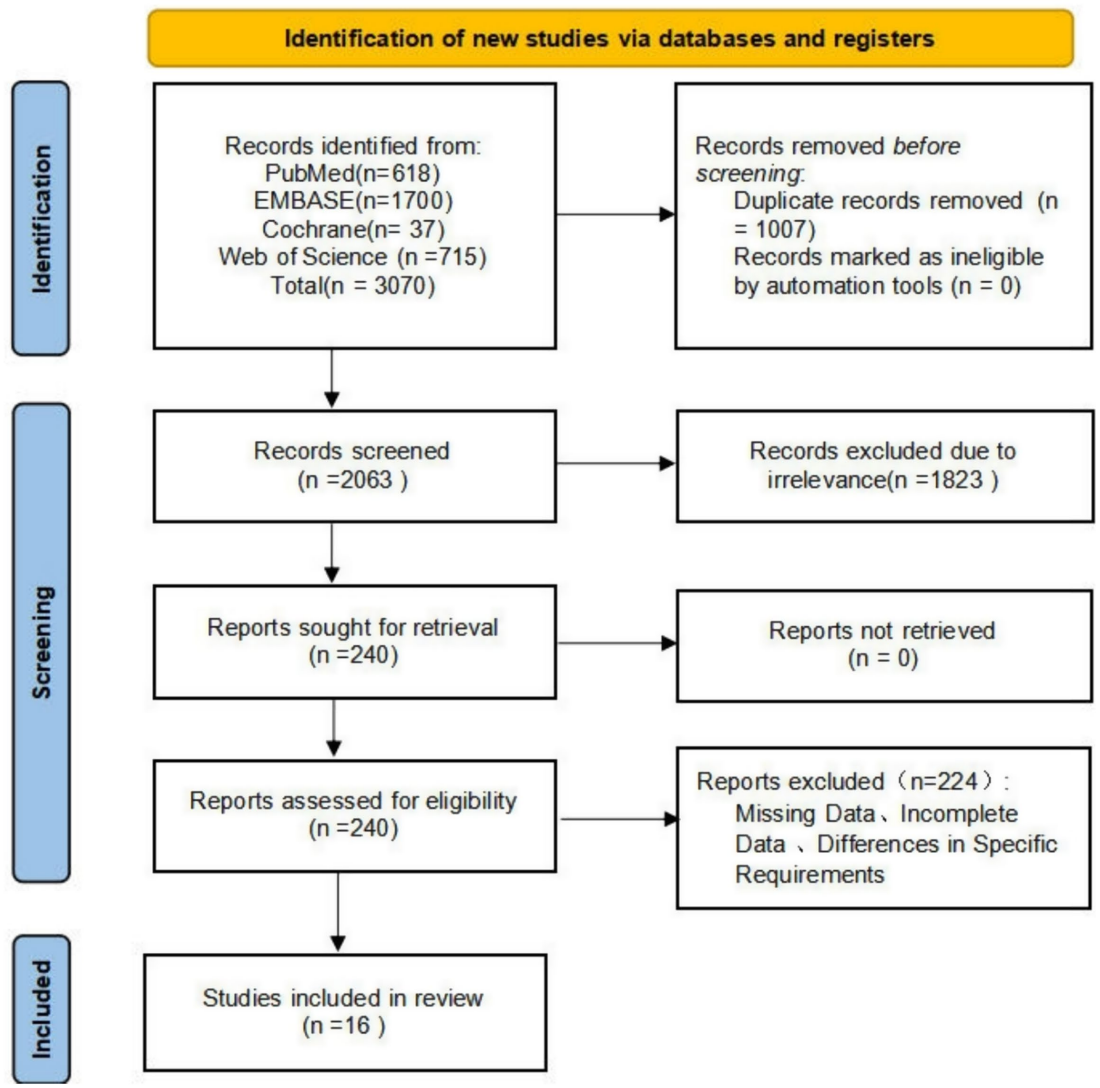
Flow diagram for study selection.

A total of 3,070 records were identified in our initial search: PubMed (*n* = 618), Embase (*n* = 1,700), Web of Science (*n* = 715), and Cochrane Library (*n* = 37). After removing duplicates and screening titles/abstracts, 240 studies were retained for full-text review. Of these, 224 did not meet the inclusion criteria and were excluded, leaving 16 studies for inclusion in our final review. Specific details are provided in Figure 1.

### 3.2 Baseline characteristics of the included literature

Following the predefined inclusion and exclusion criteria, 16 studies were ultimately included, comprising a total of 5,895 patients. Definitions of unfavorable outcome varied across studies. Thirteen studies (81.3%) used CPC 3–5 as the definition of an unfavorable outcome; one study (6.3%) used CPC 4–5; the remaining two studies adopted alternative endpoints: one used mRS 4–5, and one used the Glasgow-Pittsburgh cerebral performance classification (GCS-M) 1–3. With respect to design, 12 studies (75.0%) were prospective and four were retrospective. A summary of study characteristics is provided in Table 1.

**Table 1:** Characteristics of the included studies in the meta-analysis.

Author/year	Country	Timing of outcome assessment	Design	Primary disease	Age	Female gender	No. of patience	Main outcome	Definition of poor outcome
Backman et al. (41)	Sweden	N/A	Prospective cohort study	CA	63 (56–70)	32	207	CPC	CPC1-2 vs. CPC3-5
Scarpino et al. (42)	Italy	2016.6.1–2018.6.1	Prospective cohort study	CA	48–71	130	396	CPC	CPC1-3 vs. CPC4-5
Turella et al. (34)	Austria	N/A	Prospective cohort study	CA	65	176	873	mRS	mRS0-3 vs. mRS4-5
Admiraal et al. (35)	Netherlands	2015.4–2018.2	Prospective cohort study	CA	62	160	186	CPC	CPC1-2 vs. CPC3-5
Bang et al. (40)	South Korea	2015.10–2018.12	Prospective cohort study	CA	59	N/A	1,327	GCS-M	GCS-M4-6 vs. GCS-M1-3
Barth et al. (45)	Switzerland	2016.1–2019.3	Retrospective cohort study	CA	58/65	89	114	CPC	CPC1-2 vs. CPC3-5
Benghanem et al. (46)	France	2007–2016	Retrospective cohort study	CA	63	428	552	CPC	CPC1-2 vs. CPC3-5
Broman et al. (44)	Europe and Australia	2010–2013	Prospective cohort study	CA	66	142	169	CPC	CPC1-2 vs. CPC3-5
Glimmerveen et al. (32)	Netherlands	2010.6–2017.11	Prospective cohort study	CA	68/74	N/A	N/A	CPC	CPC1-2 vs. CPC3-5
Keijzer et al. (38)	Netherlands	2010.6–2018.6	Prospective cohort study	CA	60/65	163	683	CPC	CPC1-2 vs. CPC3-5
Kim et al. (33)	South Korea	2015.10–2018.12	Prospective cohort study	CA	58(46–69)	145	489	CPC	CPC1-2 vs. CPC3-5
Westhall et al. (43)	Sweden	2010.11–2013.1	Prospective cohort study	CA	67 ± 11	42	202	CPC	CPC1-2 vs. CPC3-5
Park et al. (39)	South Korea	2018.5–2022.6	Retrospective cohort study	CA	57(40–68)	34	130	CPC	CPC1-2 vs. CPC3-5
Rossetti et al. (21)	Switzerland	2009.4–2016.3	Prospective cohort study	CA	61.2/61.8	102	357	CPC	CPC1-2 vs. CPC3-5
Peluso et al. (37)	Belgium	2016.1–2019.3	Retrospective cohort study	CA	65 (54–72)	43	138	CPC	CPC1-2 vs. CPC3-5
Qing et al. (13)	United States	2013.11–2019.10	Prospective cohort study	CA	44.5(32–54)	23	72	CPC	CPC1-2 vs. CPC3-5

### 3.3 Quality assessment of included literature

**Figure 2:**
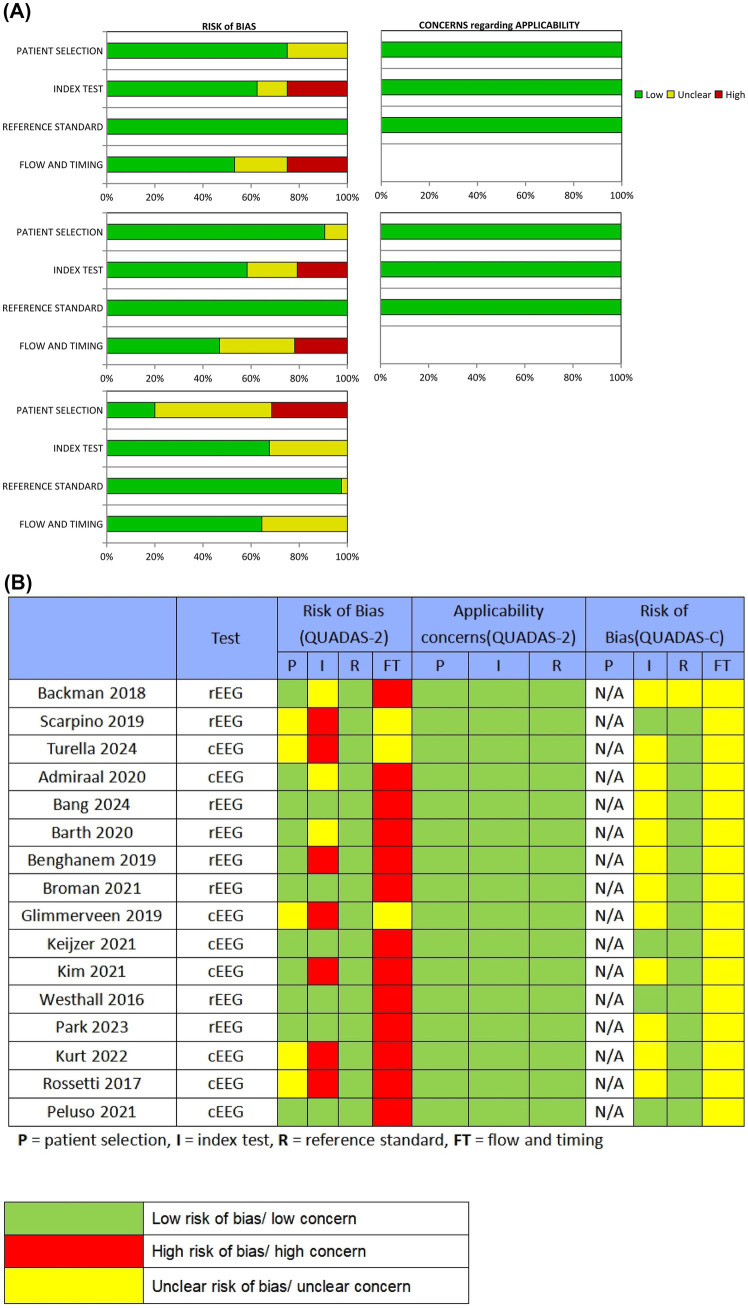
Quality assessment of the included studies. **(A)** Review authors’ judgments presented as percentages for the included studies; **(B)** Review authors’ judgments for each individual study. P = patient selection, I = index test, R = reference standard, FT = flow and timing.

Figure 2 summarize QUADAS-2 and QUADAS-C assessments, indicating mixed methodological quality. In QUADAS-2, the proportions rated with a low RoB in the cEEG subgroup were 75.0% (patient selection), 62.5% (index test), 100% (reference standard), and 53.1% (flow/timing); in the rEEG subgroup these proportions were 90.6, 58.3, 100, and 46.9%, respectively. In QUADAS-C comparative evaluations, the proportions rated with a low RoB were 20.0% (patient selection), 67.6% (index test), 97.0% (reference standard), and 64.5% (flow/timing). Major sources of RoB were evident in flow/timing, for which 46.9% (cEEG) and 53.1% (rEEG) of studies exhibited high or unclear bias. RoB was also prominent with regards to patient selection, with 80.0% of studies in QUADAS-C exhibiting high or unclear bias, largely due to nonconsecutive or nonrandom patient inclusion. Because all studies used accepted prognostic tools (CPC, mRS, or neurological examination) as primary endpoints, the reference-standard domain generally demonstrated good applicability and was rated as having a low RoB across studies.

### 3.4 Analysis of diagnostic performance

We conducted diagnostic meta-analyses on eight studies of cEEG and eight studies of rEEG; analysis indicated that both monitoring strategies exhibited high diagnostic accuracy for predicting outcomes after CA.

#### 3.4.1 Diagnostic performance of cEEG

**Figure 3:**
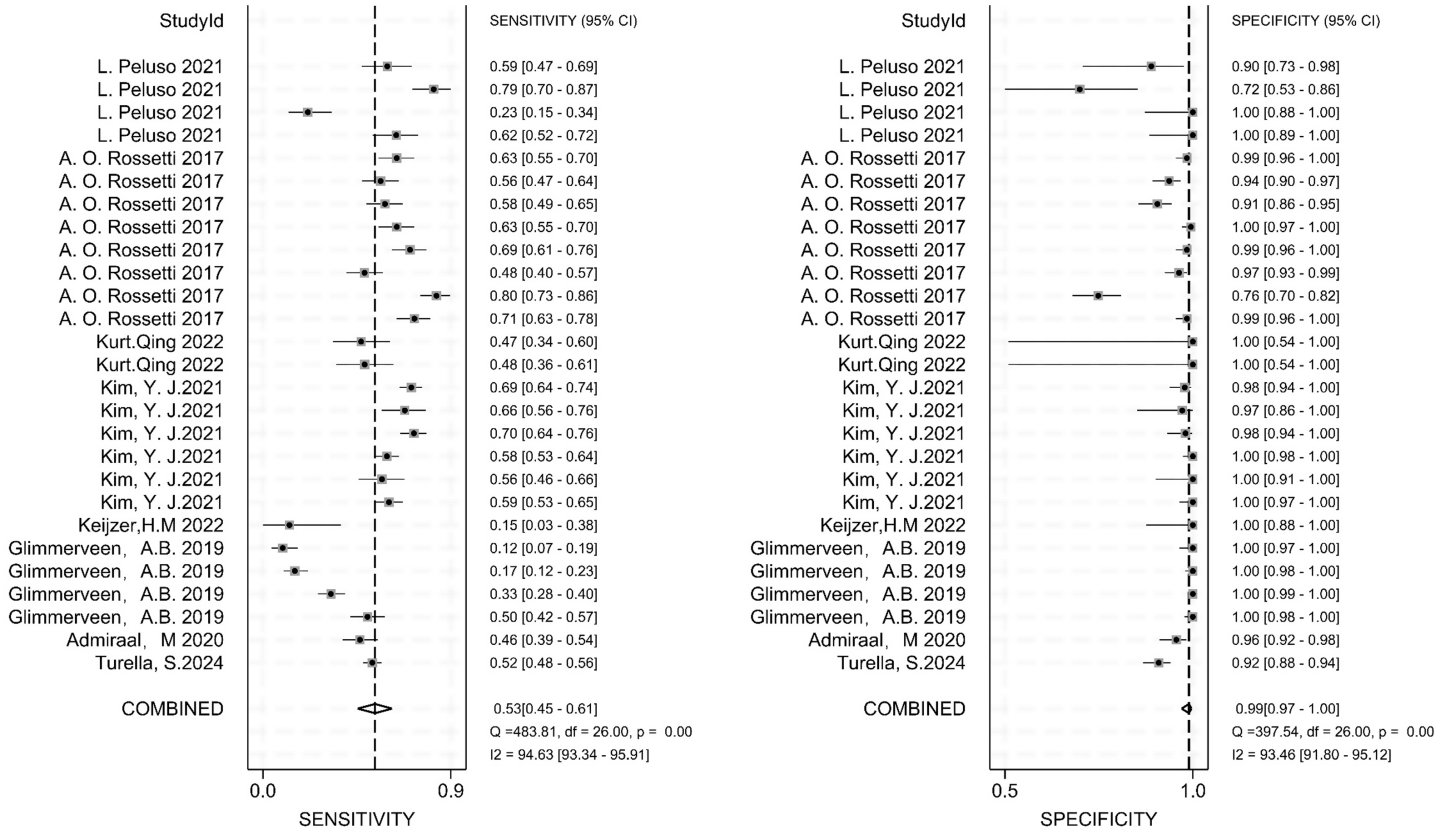
The combined sensitivity and specificity of cEEG for diagnostic purposes; cEEG, continuous electroencephalography.

Based on the pooled analysis of eight studies (13, 32–38), the combined sensitivity of cEEG for predicting unfavorable outcomes after CA was 0.53 (95% CI: 0.45–0.61; *I*^2^ = 94.63%), thus indicating moderate sensitivity for identifying patients with unfavorable outcomes. The combined specificity was 0.99 (95% CI: 0.97–1.00; *I*^2^ = 93.46%), thus suggesting extremely high accuracy for ruling out favorable outcomes. Despite substantial heterogeneity among studies (*I*^2^ > 90%), cEEG remains widely used in clinical practice for prognostic evaluation after CA (Figure 3).

#### 3.4.2 Diagnostic performance of rEEG

**Figure 4:**
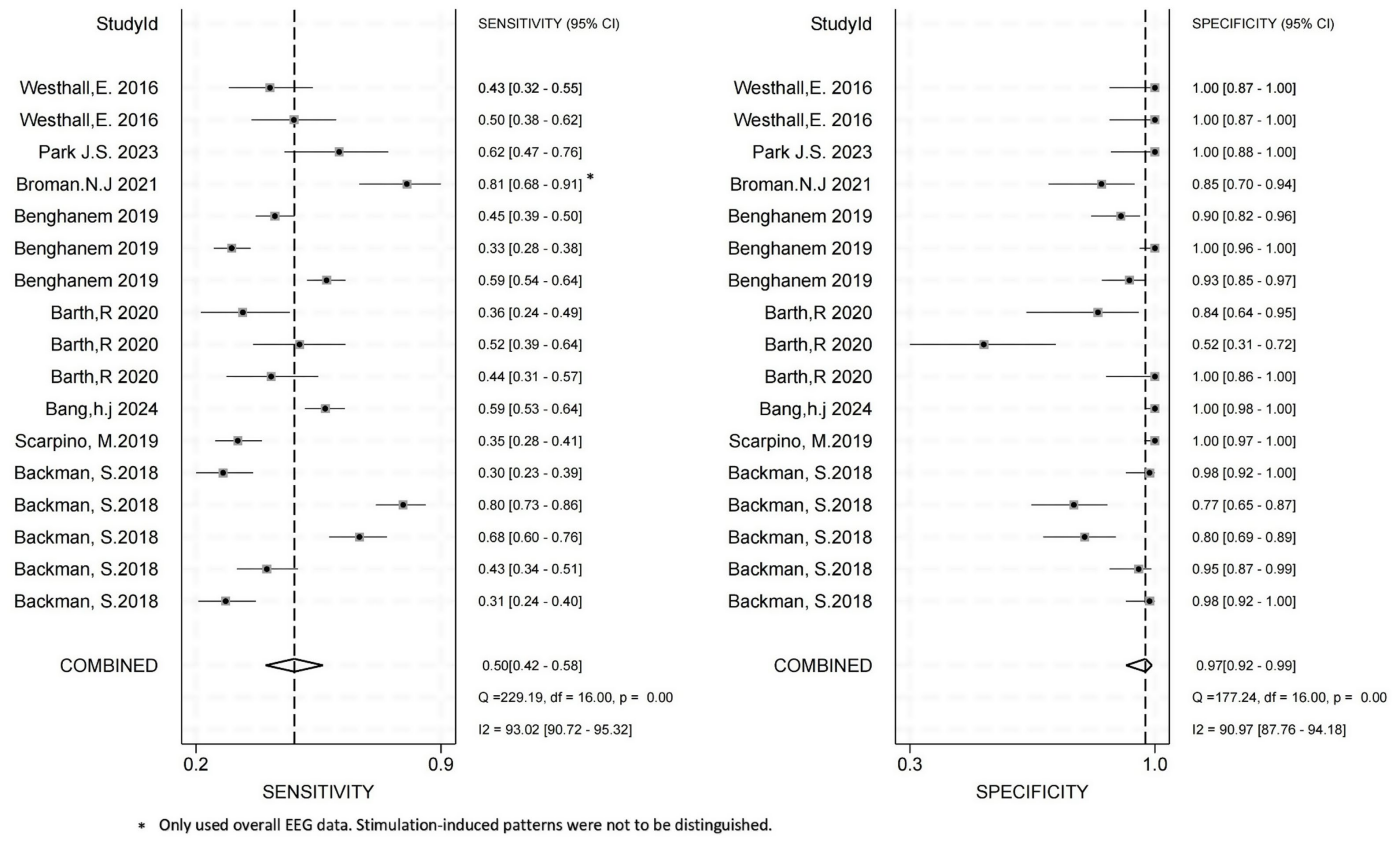
The combined sensitivity and specificity of rEEG for diagnostic purposes; rEEG, routine electroencephalography.

Based on the pooled analysis of eight studies (39–46), the diagnostic performance of rEEG for predicting unfavorable outcomes after CA had a combined sensitivity of 0.50 (95% CI: 0.42–0.58; *I*^2^ = 93.02%) and a combined specificity of 0.97 (95% CI: 0.92–0.99; *I*^2^ = 90.97%) (Figure 4).

#### 3.4.3 SROC curve analysis

**Figure 5:**
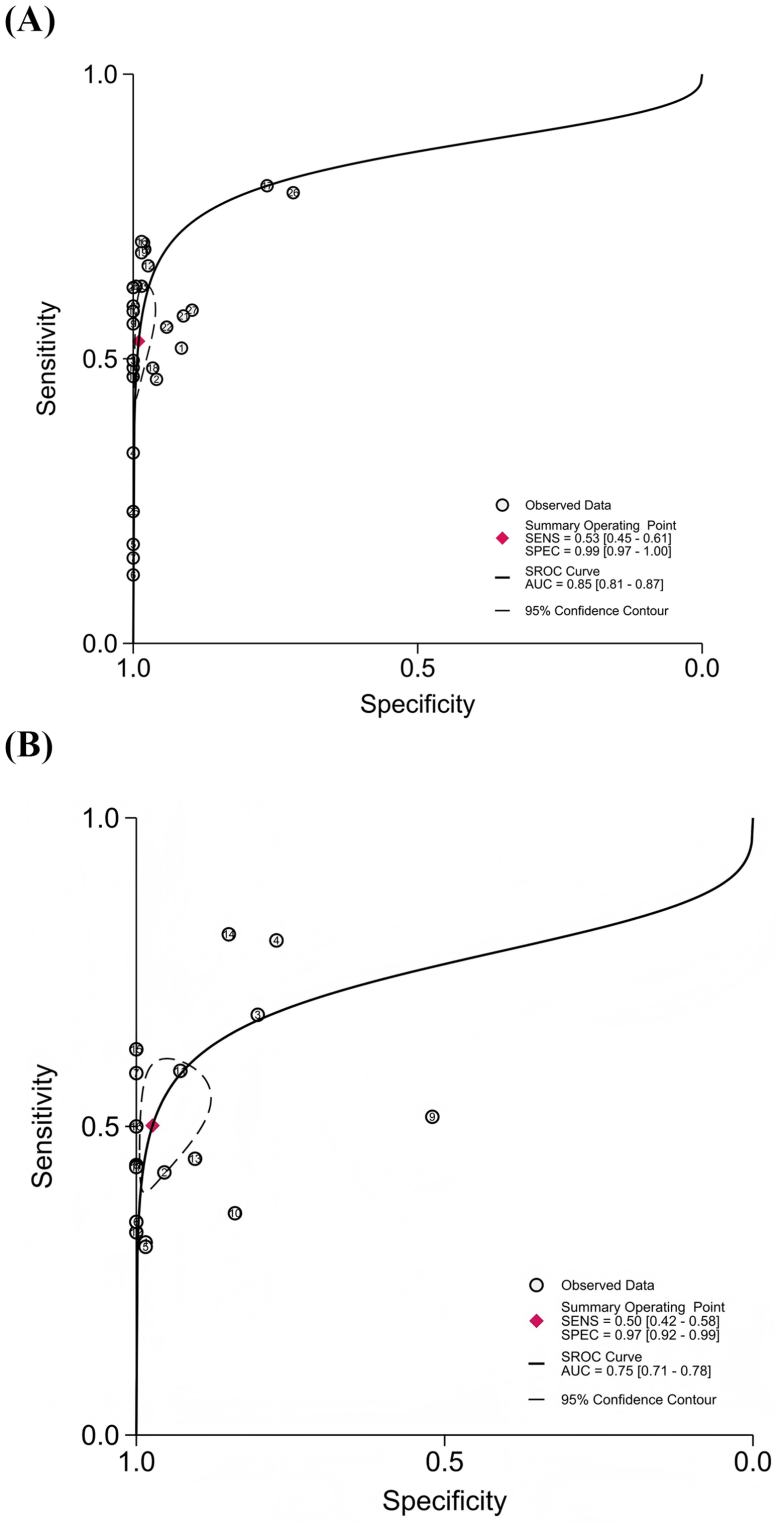
Summary receiver operating characteristic (SROC) curves for cEEG and rEEG in predicting unfavorable outcomes after cardiac arrest. **(A)** cEEG: AUC = 0.85 (95% CI: 0.81–0.87); **(B)** rEEG: AUC = 0.75 (95% CI: 0.71–0.78).

To comprehensively evaluate the overall diagnostic performance of cEEG and rEEG for the prediction of unfavorable outcomes after CA, we plotted SROC curves and calculated the AUC to reflect overall diagnostic accuracy; values closer to 1.0 indicated higher levels of performance. The AUC of the cEEG SROC curve was 0.85 (95% CI: 0.81–0.87), trending toward the upper-left corner, thus indicating good overall diagnostic accuracy. The AUC of the rEEG SROC curve was 0.75 (95% CI: 0.71–0.78), with a trajectory similar to that of cEEG, thus indicating moderately high discriminative ability (Figure 5).

### 3.5 Assessment of publication bias

**Figure 6:**
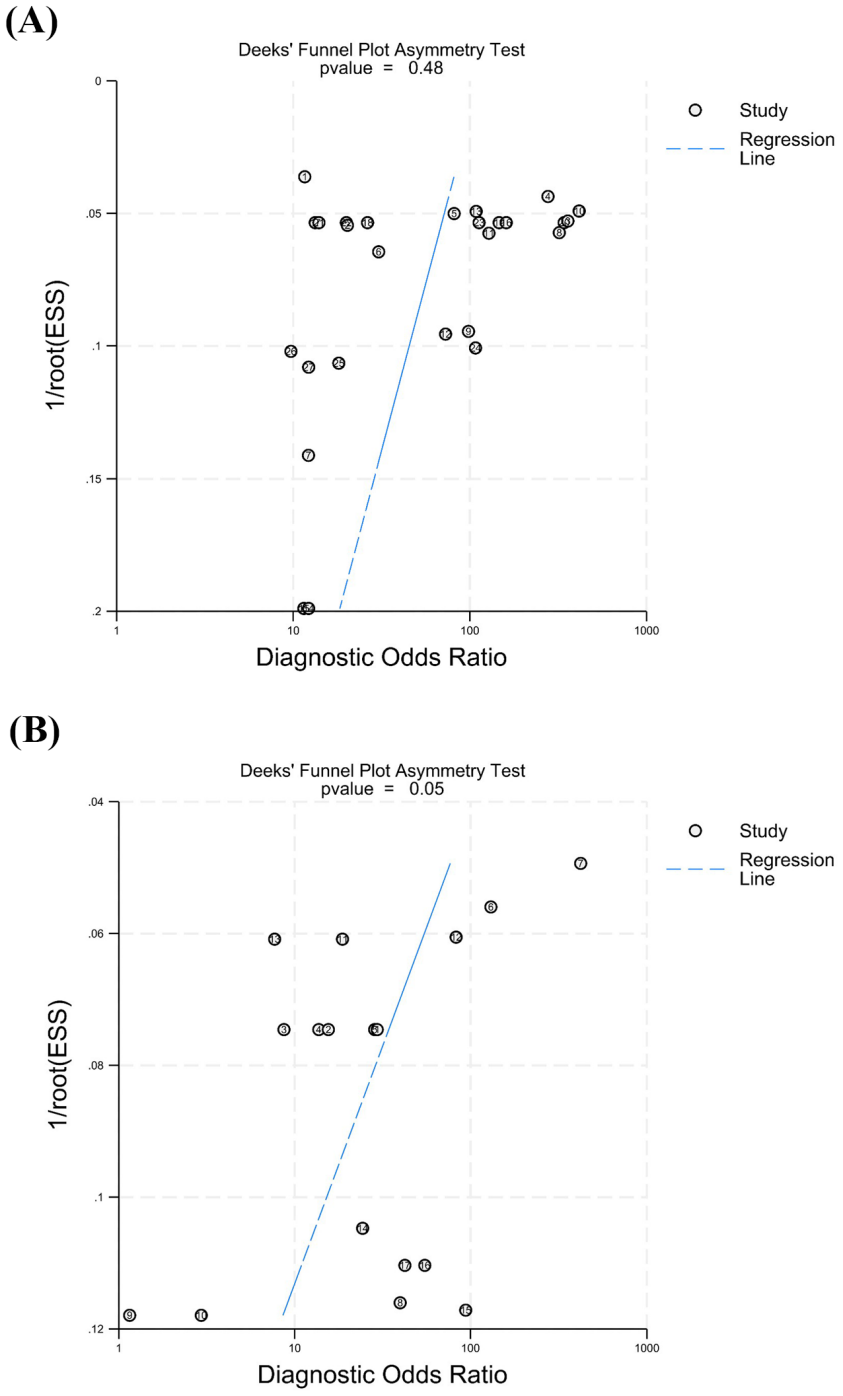
The Deeks’ test was performed to assess publication bias. **(A)** For cEEG, the *p* value was 0.48 (*p* > 0.05), indicating no significant publication bias. **(B)** For rEEG, the *p* value was 0.05 (*p* = 0.05), suggesting mild publication bias.

Publication bias was assessed using Deeks’ test based on the studies included in this meta-analysis (Figure 6). The *p* values were 0.48 for cEEG (*p* > 0.05) and 0.05 for rEEG (*p* = 0.05), thus indicating no significant publication bias for cEEG and mild publication bias for rEEG.

### 3.6 Sensitivity analysis

**Figure 7:**
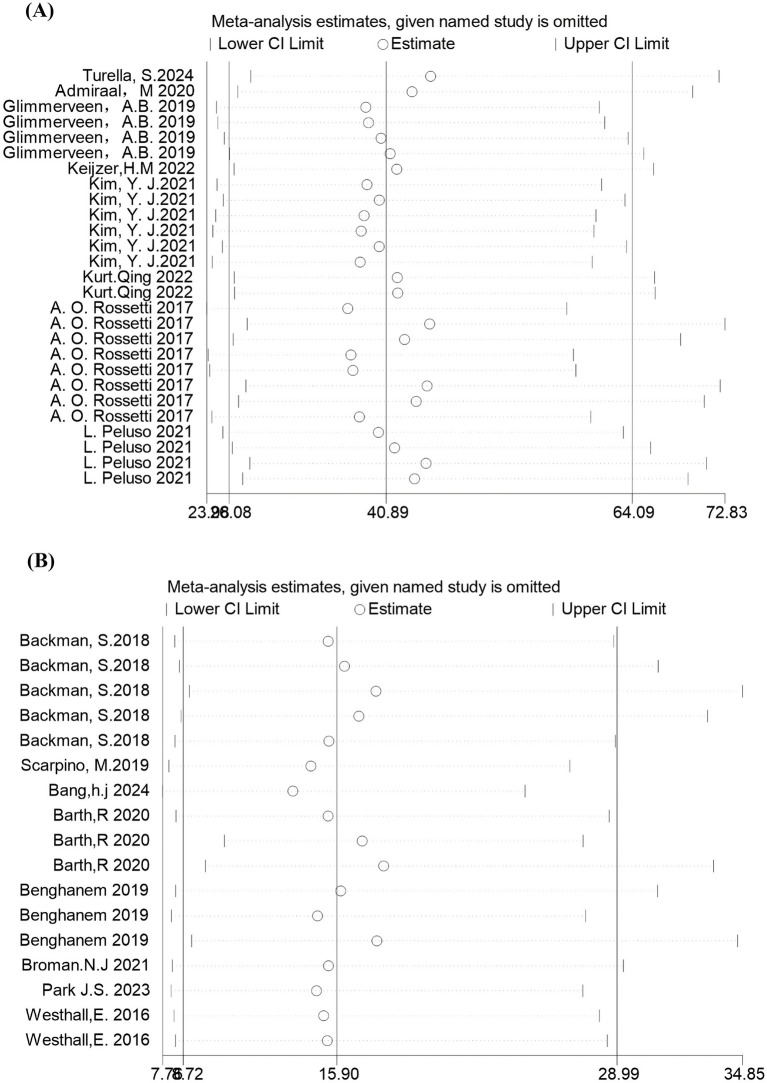
Sensitivity analysis of diagnostic odds ratio (DOR) by sequential exclusion of individual studies. **(A)** cEEG: pooled DOR = 40.89 (95% CI: 26.08–64.09); **(B)** rEEG: pooled DOR = 15.90 (95% CI: 8.72–28.99). The consistent estimates after omitting each study indicate robust results.

Sensitivity analyses revealed a pooled effect size for DOR of 40.89 (95% CI: 26.08–64.09) for cEEG and a DOR of 15.90 (95% CI: 8.72–28.99) for rEEG, thus indicating comparable diagnostic performance with robust results. The 95% CIs partially overlapped, suggesting no significant difference in predictive value. After sequentially omitting individual studies, point estimates remained within the overall CIs, thus indicating that conclusions did not depend on any single study (Figure 7).

## 4 Discussion

EEG is among the most extensively used prognostic tool complementing bedside clinical examination, as this technique enables the specific prediction of neurological outcomes by detecting abnormal neuronal activity (47–49). Prognostic evaluation after CA is central to clinical decision-making and can directly influence therapeutic strategies and informed consent. As an evaluative “gold standard” for post-CA brain function, cEEG has been increasingly used for neuro-monitoring in critically ill patients (47); the high specificity of this technique confers irreplaceable value in the diagnosis of poor prognosis. However, the broad implementation of cEEG is limited by high cost, resource intensity, and low availability, preventing many patients from timely cEEG assessment. In this context, whether the more convenient and economical rEEG can achieve a diagnostic performance comparable to cEEG has become a pressing clinical question.

With increasing attention to post-CA neuroprognostication, the value of rEEG has been actively explored (50–52), yet findings remain inconsistent. To systematically evaluate and clarify the diagnostic performance and clinical utility of cEEG and rEEG, we conducted a meta-analysis of the literature to comprehensively assess both modalities for predicting unfavorable outcomes after CA.

Our meta-analysis revealed that cEEG had a pooled sensitivity of 0.53, a specificity of 0.99, and an AUC of 0.85. In contrast, rEEG had a pooled sensitivity of 0.50, a specificity of 0.97, and an AUC of 0.75. Collectively, these results indicate that both EEG modalities exhibited very high specificity, supporting their reliability for ruling out favorable outcomes and assisting clinical decision-making. However, both techniques demonstrated only moderate sensitivity, highlighting limitations in the identification of patients with unfavorable outcomes. From an overall perspective, both AUCs exceeded the 0.75 threshold, thus indicating good discriminative capacity. Notably, rEEG exhibited comparable pooled sensitivity to cEEG and can be completed over a shorter time without prolonged monitoring. This advantage affords an overall diagnostic performance for rEEG that is similar to cEEG in terms of rapid prognostic evaluation, thus suggesting its potential as a practical alternative in settings requiring prompt decisions or with limited monitoring resources.

Sensitivity analyses yielded an DOR of 40.89 for cEEG and an DOR of 15.90 for rEEG, with partially overlapping 95% CIs, thus indicating broadly similar overall effects and no significant difference in diagnostic performance. Publication-bias analysis yielded *p* = 0.48 for cEEG according to Deeks’ test, suggesting no significant bias, and *p* = 0.05 for rEEG, thus suggesting mild bias. Given the considerable heterogeneity across studies, including differences in patient populations, EEG acquisition windows, monitoring duration, interpretive criteria, clinical management, and statistical methods, such bias signal, may partly reflect methodological variation. Despite a mild bias signal for rEEG, sensitivity analyses indicated stable effect sizes and diagnostic performance comparable to cEEG, thus supporting the robustness of our findings.

This meta-analysis has limitations that need to be considered. First, heterogeneity among the included studies was substantial. Potential sources include variability in outcome definitions and thresholds, study designs and patient cohorts, EEG monitoring strategies and procedures, clinical management confounders, and statistical approaches, all of which may contribute to variability in our results. Second, direct head-to-head comparisons of cEEG and rEEG remain scarce; current evidence relies predominantly on indirect comparisons across different studies. Because insufficient research has applied both methods concurrently within the same cohort, we were unable to directly assess differences in diagnostic performance under identical clinical conditions, and residual confounding by population or setting cannot be ruled out. Accordingly, more rigorously designed diagnostic-accuracy studies employing direct comparisons are now needed to further investigate the relative value of cEEG and rEEG in post-CA prognostication.

In summary, rEEG demonstrates a core diagnostic performance that is highly consistent with that of cEEG for the prediction of unfavorable outcomes after CA. These findings provide key evidence to guide the rational selection of neuroprognostic monitoring strategies in resource-limited or rapid-assessment contexts, with important implications for optimizing resource allocation, expanding EEG use in neurocritical evaluation, and improving outcomes for patients after CA. rEEG may serve as an effective alternative or complement to cEEG and play a central role in broader clinical practice.

## 5 Nomenclature

### 5.1 Resource identification initiative

As this study is a systematic review and meta-analysis, no primary wet-lab reagents, cell lines, model organisms, or other physical biological resources were used or generated; therefore, there are no Research Resource Identifiers to report. To ensure reproducibility of the analyses, we provide detailed identification of all included studies (including original-study DOIs, PubMed IDs, or other database identifiers), the search strategy, inclusion/exclusion criteria, data extraction procedures, and the code and software versions used for statistical analyses; these materials and code are archived in a public repository with permanent links/DOIs for access and replication.

### 5.2 Life science identifiers

This manuscript does not involve taxonomic naming acts or registrations; therefore, no life science identifiers (LSIDs) are provided. If LSID-bearing records are cited in future work, we will include the relevant LSIDs and registry links in the appropriate sections and Supplementary materials as required.

## Data Availability

The original contributions presented in the study are included in the article/Supplementary material, further inquiries can be directed to the corresponding author/s.

## Glossary

cEEG: Continuous Electroencephalography
rEEG: Routine Electroencephalography
QUADAS-2: Quality Assessment of Diagnostic Accuracy Studies 2
QUADAS-C: Quality Assessment of Diagnostic Accuracy Studies-C
SROC: Summary Receiver Operating Characteristic
AUC: Area under the SROC Curve
CI: Confidence Intervals
CA: Cardiac Arrest
ROSC: Return of Spontaneous Circulation
CPC: The Cerebral Performance Category
NCSE: Nonconvulsive Status Epilepticus
VF: Ventricular Fibrillation
PEA: Pulseless Electrical Activity
EEG: Electroencephalography
NSE: Neuron-specific Enolase
ACNS: The American Clinical Neurophysiology Society
PRISMA: Preferred Reporting Items for Systematic Reviews and Meta-Analyses
GCS: Glasgow Coma Score
TP: True Positives
FP: False Positives
FN: False Negatives
TN: True Negatives
RoB: Risk of bias
DOR: Diagnostic Odds Ratio
CI: The Confidence Intervals.
