# Supplementary material for: cEEG and rEEG detection rates of prognostic indicators in cardiac arrest patients: a systematic review and diagnostic meta-analysis
Source: Front Neurol. 2026 Feb 18;17:1760363. doi: 10.3389/fneur.2026.1760363 (PMC12956694; doi:10.3389/fneur.2026.1760363)
Supplement: Supplementary file 1 [file Table_1.docx]

**SUPPLEMENTARY MATERIALS**

[Supplementary Table 1 Preferred Reporting Items for Systematic Reviews and Meta‑Analyses (PRISMA) guidelines for diagnostic test accuracy reviews 2](#_Toc2639)

[Supplementary Table 2 The search strategy of this study (PubMed Example) 4](#_Toc29696)

[Supplementary Table 3 The modified Quality Assessment of Diagnostic Accuracy Studies-2 (QUADAS-2) checklist used for risk of bias assessment and applicability concern  5](#_Toc9474)

[Supplementary Table 4 Overview of Data Extracted from the Included Studies 9](#_Toc18156)

**Supplementary Table 1** Preferred Reporting Items for Systematic Reviews and Meta‑Analyses (PRISMA) guidelines for diagnostic test accuracy reviews

| **Section and Topic** | **Item #** | **Checklist item** | **Location where item is reported** |
| --- | --- | --- | --- |
| **TITLE** | | |  |
| Title | 1 | Identify the report as a systematic review. | Title |
| **ABSTRACT** | | |  |
| Abstract | 2 | See the PRISMA 2020 for Abstracts checklist. | Abstract |
| **INTRODUCTION** | | |  |
| Rationale | 3 | Describe the rationale for the review in the context of existing knowledge. | Part 1 |
| Objectives | 4 | Provide an explicit statement of the objective(s) or question(s) the review addresses. | Part 1 |
| **METHODS** | | |  |
| Eligibility criteria | 5 | Specify the inclusion and exclusion criteria for the review and how studies were grouped for the syntheses. | Part 2.1.1 |
| Information sources | 6 | Specify all databases, registers, websites, organisations, reference lists and other sources searched or consulted to identify studies. Specify the date when each source was last searched or consulted. | Part 2.2 |
| Search strategy | 7 | Present the full search strategies for all databases, registers and websites, including any filters and limits used. | Part 2.2 |
| Selection process | 8 | Specify the methods used to decide whether a study met the inclusion criteria of the review, including how many reviewers screened each record and each report retrieved, whether they worked independently, and if applicable, details of automation tools used in the process. | Part 2.2 |
| Data collection process | 9 | Specify the methods used to collect data from reports, including how many reviewers collected data from each report, whether they worked independently, any processes for obtaining or confirming data from study investigators, and if applicable, details of automation tools used in the process. | Part 2.3 |
| Data items | 10a | List and define all outcomes for which data were sought. Specify whether all results that were compatible with each outcome domain in each study were sought (e.g. for all measures, time points, analyses), and if not, the methods used to decide which results to collect. | Part 2.3 |
|  | 10b | List and define all other variables for which data were sought (e.g. participant and intervention characteristics, funding sources). Describe any assumptions made about any missing or unclear information. | Part 2.1.2, 2.1.3, 2.1.4, 2.1.5 |
| Study risk of bias assessment | 11 | Specify the methods used to assess risk of bias in the included studies, including details of the tool(s) used, how many reviewers assessed each study and whether they worked independently, and if applicable, details of automation tools used in the process. | Part 2.3 |
| Effect measures | 12 | Specify for each outcome the effect measure(s) (e.g. risk ratio, mean difference) used in the synthesis or presentation of results. | Part 2.4 |
| Synthesis methods | 13a | Describe the processes used to decide which studies were eligible for each synthesis (e.g. tabulating the study intervention characteristics and comparing against the planned groups for each synthesis (item #5)). | Part 2.1, 2.2, 2.4 |
|  | 13b | Describe any methods required to prepare the data for presentation or synthesis, such as handling of missing summary statistics, or data conversions. | Part 2.3, 2.4, 2.5 |
|  | 13c | Describe any methods used to tabulate or visually display results of individual studies and syntheses. | Part 2.3, 2.4 |
|  | 13d | Describe any methods used to synthesize results and provide a rationale for the choice(s). If meta-analysis was performed, describe the model(s), method(s) to identify the presence and extent of statistical heterogeneity, and software package(s) used. | Part 2.4 |
|  | 13e | Describe any methods used to explore possible causes of heterogeneity among study results (e.g. subgroup analysis, meta-regression). | Part 2.4, 2.5 |
|  | 13f | Describe any sensitivity analyses conducted to assess robustness of the synthesized results. | Part 2.4, 2.5 |
| Reporting bias assessment | 14 | Describe any methods used to assess risk of bias due to missing results in a synthesis (arising from reporting biases). | Part 2.4 |
| Certainty assessment | 15 | Describe any methods used to assess certainty (or confidence) in the body of evidence for an outcome. | Part 2.3, 2.4 |
| **RESULTS** | | |  |
| Study selection | 16a | Describe the results of the search and selection process, from the number of records identified in the search to the number of studies included in the review, ideally using a flow diagram. | Part 3.1 |
|  | 16b | Cite studies that might appear to meet the inclusion criteria, but which were excluded, and explain why they were excluded. | Part 3.1 |
| Study characteristics | 17 | Cite each included study and present its characteristics. | Part 3.2 |
| Risk of bias in studies | 18 | Present assessments of risk of bias for each included study. | Part 3.3 |
| Results of individual studies | 19 | For all outcomes, present, for each study: (a) summary statistics for each group (where appropriate) and (b) an effect estimate and its precision (e.g. confidence/credible interval), ideally using structured tables or plots. | Part 3.4 |
| Results of syntheses | 20a | For each synthesis, briefly summarise the characteristics and risk of bias among contributing studies. | Part 3.2, 3.3 |
|  | 20b | Present results of all statistical syntheses conducted. If meta-analysis was done, present for each the summary estimate and its precision (e.g. confidence/credible interval) and measures of statistical heterogeneity. If comparing groups, describe the direction of the effect. | Part 3.4, 3.6 |
|  | 20c | Present results of all investigations of possible causes of heterogeneity among study results. | Part 4 |
|  | 20d | Present results of all sensitivity analyses conducted to assess the robustness of the synthesized results. | Part 3.6 |
| Reporting biases | 21 | Present assessments of risk of bias due to missing results (arising from reporting biases) for each synthesis assessed. | Part 3.5 |
| Certainty of evidence | 22 | Present assessments of certainty (or confidence) in the body of evidence for each outcome assessed. | Part 3.3, 3.4, 3.5, 3.6 |
| **DISCUSSION** | | |  |
| Discussion | 23a | Provide a general interpretation of the results in the context of other evidence. | Part 4 |
|  | 23b | Discuss any limitations of the evidence included in the review. | Part 4 |
|  | 23c | Discuss any limitations of the review processes used. | Part 4 |
|  | 23d | Discuss implications of the results for practice, policy, and future research. | Part 4 |
| **OTHER INFORMATION** | | |  |
| Registration and protocol | 24a | Provide registration information for the review, including register name and registration number, or state that the review was not registered. | Abstract |
|  | 24b | Indicate where the review protocol can be accessed, or state that a protocol was not prepared. | Abstract |
|  | 24c | Describe and explain any amendments to information provided at registration or in the protocol. | Abstract |
| Support | 25 | Describe sources of financial or non-financial support for the review, and the role of the funders or sponsors in the review. | Part 8 |
| Competing interests | 26 | Declare any competing interests of review authors. | Part 6 |
| Availability of data, code and other materials | 27 | Report which of the following are publicly available and where they can be found: template data collection forms; data extracted from included studies; data used for all analyses; analytic code; any other materials used in the review. | Part 11 |

**Supplementary Table 2** The search strategy of this study (PubMed Example)

|  | Search number | Query |
| --- | --- | --- |
| Patient (P) | 1 | (Heart arrest) OR (Cardiopulmonary Resuscitation) OR (Heart Massage) OR (Death (sudden cardiac)) OR (Cardiac arrest) OR (Cardiopulmonary Arrest) OR (Asystole*) OR (Cardiac Resuscitation*) OR (Heart Massage*) OR (Cardiac Massage*) |
|  | 2 | (Hypoxia-Ischemia, Brain) OR {[(Hypoxic-Ischemic) OR (Ischemic-Hypoxic) OR (Hypoxia-Ischemia*) OR (Ischemia-Hypoxia*) OR (Anoxic-Ischemic) OR (Anoxia-Ischemia*) OR (Ischemia-Anoxia*)] AND ((Brain) OR (Encephalopath*) OR (Cerebral))} |
|  | 3 | (Coma) OR (Comas) OR (Comatose) |
|  | 4 | #2 OR #3 |
| Intervention (I) | 5 | (cEEG) OR (Continuous EEG) OR (Continuous electroencephalogram) OR (rEEG) OR (Routine EEG) OR (Routine electroencephalogram) OR (EEG) OR (electroencephalogram) OR (Electroencephalography) OR (Consciousness Monitors) OR (electrophysiolog*) OR (Consciousness Monitor*) OR (Index of Consciousness-View Monitor*) OR (Index of ConsciousnessView Monitor*) OR (Bispectral Index Monitor*) OR (Bispectral Index Monitor*) OR (spectral array) |
|  | 6 | ("2010/1/1"[Date - Publication] : "2024/12/31"[Date - Publication]) |
| P I | 7 | #1 AND #4 AND #5 AND #6 |

# **Supplementary Table 3** The modified Quality Assessment of Diagnostic Accuracy Studies-2 (QUADAS-2) checklist used for risk of bias assessment and applicability concern

| **Domain 1: Patient Selection** | | | |
| --- | --- | --- | --- |
| Information to support judgment | *Describe methods of patient selection.*  *Describe included patients (previous testing, presentation, intended use of index test, and setting).*  *Describe how patients were allocated to receive each of the index tests. If randomization was used to assign individual patients (or clusters of patients) to index tests, describe the randomization process.* | | |
| **Single test accuracy (QUADAS-2)** | | **Answers for cEEG** | **Answers for rEEG** |
| Signaling questions | 1.1 Was a consecutive or random sample of patients enrolled? | Yes/No/Unclear | Yes/No/Unclear |
|  | 1.2 Was a case-control design avoided? | Yes/No/Unclear | Yes/No/Unclear |
|  | 1.3 Did the study avoid inappropriate exclusions? | Yes/No/Unclear | Yes/No/Unclear |
| Risk of bias | 1.4 Could the selection of patients have introduced bias? | Low/High/Unclear | Low/High/Unclear |
| Concerns regarding applicability | 1.5 Are there concerns that the included patients do not match the review question? | Low/High/Unclear | Low/High/Unclear |
| **Comparative accuracy (QUADAS-C)** | | **Answers for the**  **test comparison** | |
| Signaling questions | C1.1 Was the risk of bias for each index test judged ‘low’ for this domain? | Yes/No | |
|  | C1.2 Was a fully paired or randomized design used? | Yes/No/Unclear | |
|  | C1.3 Was the allocation sequence random? | Yes/No/Unclear/ Not applicable | |
|  | C1.4 Was the allocation sequence concealed until patients were enrolled and assigned to index tests?† | Yes/No/Unclear/ Not applicable | |
| Risk of bias | C1.5 Could the selection of patients have introduced bias in the comparison? | Low/High/Unclear | |

*† Only applicable to randomized designs.*

| **Domain 2: Index Test** | | | |
| --- | --- | --- | --- |
| Information to support judgment | *Describe the index tests and how they were conducted and interpreted.*  *For paired comparative studies, describe the order in which the index tests were performed.* | | |
| **Single test accuracy (QUADAS-2)** | | **Answers for cEEG** | **Answers for rEEG** |
| Signaling questions | 2.1 Were the index test results interpreted without knowledge of the results of the reference standard? | Yes/No/Unclear | Yes/No/Unclear |
|  | 2.2 If a threshold was used, was it prespecified? | Yes/No/Unclear | Yes/No/Unclear |
| Risk of bias | 2.3 Could the conduct or interpretation of the index test have introduced bias? | Low/High/Unclear | Low/High/Unclear |
| Concerns regarding applicability | 2.4 Are there concerns that the index test, its conduct or its interpretation differ from the review question? | Low/High/Unclear | Low/High/Unclear |
| **Comparative accuracy (QUADAS-C)** | | **Answers for the**  **test comparison** | |
| Signaling questions | C2.1 Was the risk of bias for each index test judged ‘low’ for this domain? | Yes/No | |
|  | C2.2 Were the index test results interpreted without knowledge of the results of the other index test(s)?‡ | Yes/No/Unclear/ Not applicable | |
|  | C2.3 Is undergoing one index test unlikely to affect the performance of the other index test(s)?‡ | Yes/No/Unclear/ Not applicable | |
|  | C2.4 Were the index tests conducted and interpreted without advantaging one of the tests? | Yes/No/Unclear | |
| Risk of bias | C2.5 Could the conduct or interpretation of the index tests have introduced bias in the comparison? | Low/High/Unclear | |

*‡ Only applicable if patients received multiple index tests (fully or partially paired designs)*

| **Domain 3: Reference Standard** | | | |
| --- | --- | --- | --- |
| Information to support judgment | *Describe the reference standard, how it was conducted and interpreted, and whether any of the index tests were part of the reference standard.* | | |
| **Single test accuracy (QUADAS-2)** | | **Answers for cEEG** | **Answers for rEEG** |
| Signaling questions | 3.1 Is the reference standard likely to correctly classify the target condition? | Yes/No/Unclear | Yes/No/Unclear |
|  | 3.2 Were the reference standard results interpreted without knowledge of the results of the index test? | Yes/No/Unclear | Yes/No/Unclear |
| Risk of bias | 3.3 Could the reference standard, its conduct, or its interpretation have introduced bias? | Low/High/Unclear | Low/High/Unclear |
| Concerns regarding applicability | 3.4 Are there concerns that the target condition as defined by the reference standard does not match the review question? | Low/High/Unclear | Low/High/Unclear |
| **Comparative accuracy (QUADAS-C)** | | **Answers for the**  **test comparison** | |
| Signaling questions | C3.1 Was the risk of bias for each index test judged ‘low’ for this domain? | Yes/No | |
|  | C3.2 Did the reference standard avoid incorporating any of the index tests? | Yes/No/Unclear | |
| Risk of bias | C3.3 Could the reference standard, its conduct, or its interpretation have introduced bias in the comparison? | Low/High/Unclear | |

| **Domain 4: Flow and Timing** | | | |
| --- | --- | --- | --- |
| Information to support judgment | *Describe any patients who did not receive the index tests or reference standard or who were excluded from the analysis.*  *Describe the time interval and any interventions between the index tests and the reference standard.*  *Describe the time interval and any interventions between the index tests being compared.* | | |
| **Single test accuracy (QUADAS-2)** | | **Answers for cEEG** | **Answers for rEEG** |
| Signaling questions | 4.1 Was there an appropriate interval between index tests and reference standard? | Yes/No/Unclear | Yes/No/Unclear |
|  | 4.2 Did all patients receive a reference standard? | Yes/No/Unclear | Yes/No/Unclear |
|  | 4.3 Did all patients receive the same reference standard? | Yes/No/Unclear | Yes/No/Unclear |
|  | 4.4 Were all patients included in the analysis? | Yes/No/Unclear | Yes/No/Unclear |
| Risk of bias | 4.5 Could the patient flow have introduced bias? | Low/High/Unclear | Low/High/Unclear |
| **Comparative accuracy (QUADAS-C)** | | **Answers for the**  **test comparison** | |
| Signaling questions | C4.1 Was the risk of bias for each index test judged ‘low’ for this domain? | Yes/No | |
|  | C4.2 Was there an appropriate interval between the index tests? | Yes/No/Unclear | |
|  | C4.3 Was the same reference standard used for all index tests? | Yes/No/Unclear | |
|  | C4.4 Are the proportions and reasons for missing data similar across index tests? | Yes/No/Unclear | |
| Risk of bias | C4.5 Could the patient flow have introduced bias in the comparison? | Low/High/Unclear | |

**Supplementary Table 4** Overview of Data Extracted from the Included Studies

| **Author** | **Year** | **poor outcome** | **Definition** | **TP** | **FP** | **FN** | **TN** | **Data Source** | **Handling of Multiple Data** | **Reviewer 1** | **Reviewer 2** | **Consensus** |
| --- | --- | --- | --- | --- | --- | --- | --- | --- | --- | --- | --- | --- |
| **Backman,S.** | 2018 | CPC1-2 vs CPC3-5 | rEEG | 44 | 1 | 97 | 65 | Table 2 | Highly malignant pattern | LJY | YYH | Approved |
|  |  |  |  | 60 | 3 | 81 | 63 |  | Malignant rhythmic or periodic features |  |  |  |
|  |  |  |  | 96 | 13 | 45 | 53 |  | Malignant background |  |  |  |
|  |  |  |  | 113 | 15 | 28 | 51 |  | At least one malignant feature |  |  |  |
|  |  |  |  | 43 | 1 | 98 | 65 |  | Both malignant background and rhythmic/ periodic features |  |  |  |
| **Scarpino,M.** | 2019 | CPC1-3 vs CPC4-5 | rEEG | 77 | 0 | 146 | 123 | Table 2 | N/A | LX | JZH | Approved |
| **Turella,S.** | 2024 | mRS0-3 vs mRS4-5 | cEEG | 306 | 24 | 284 | 259 | Table 2 | N/A | YYH | LJY | Approved |
| **Admiraal,M** | 2020 | CPC1-2 vs CPC3-5 | cEEG | 77 | 7 | 89 | 164 | Figure3 | N/A | YYH | LJY | Approved |
| **Bang,h.j** | 2024 | GCS-M4-6 vs GCS-M1-3 | rEEG | 190 | 0 | 134 | 149 | Table 2 | N/A | LX | JZH | Approved |
| **Barth,R** | 2020 | CPC1-2 vs CPC3-5 | rEEG | 28 | 0 | 36 | 25 | Table 1 | EEG highly malignant pattern | JZH | LX | Approved |
|  |  |  |  | 33 | 12 | 31 | 13 |  | EEG malignant pattern |  |  |  |
|  |  |  |  | 23 | 4 | 41 | 21 |  | EEG epileptiform activity |  |  |  |
| **Benghanem** | 2019 | CPC1-2 vs CPC3-5 | rEEG | 203 | 6 | 141 | 78 | Table 2 | Non-reactive EEG | YYH | LJY | Approved |
|  |  |  |  | 113 | 0 | 231 | 84 |  | Highly malignant pattern |  |  |  |
|  |  |  |  | 154 | 8 | 190 | 76 |  | Malignant pattern |  |  |  |
| **Glimmerveen,A.B.** | 2019 | CPC1-2 vs CPC3-5 | cEEG | 87 | 0 | 88 | 181 | Table 1 | at 12 h | LJY | YYH | Approved |
|  |  |  |  | 84 | 0 | 167 | 274 |  | at 24 h |  |  |  |
|  |  |  |  | 36 | 0 | 171 | 191 |  | at 36 h |  |  |  |
|  |  |  |  | 16 | 0 | 117 | 108 |  | at 48 h |  |  |  |
| **Kim, Y. J.** | 2021 | CPC1-2 vs CPC3-5 | cEEG | 144 | 0 | 99 | 110 | Table 4 | Highly malignant pattern < 72 h | JZH | LX | Approved |
|  |  |  |  | 55 | 0 | 43 | 38 |  | Highly malignant pattern between 72 h and 7 days |  |  |  |
|  |  |  |  | 199 | 0 | 142 | 148 |  | Highly malignant pattern < 7 days |  |  |  |
|  |  |  |  | 171 | 2 | 72 | 108 |  | Highly malignant or malignant patterns with delta or undetermined frequency < 72 h |  |  |  |
|  |  |  |  | 65 | 1 | 33 | 37 |  | Highly malignant or malignant patterns with delta or undetermined frequency between 72 h and 7 days |  |  |  |
|  |  |  |  | 236 | 3 | 105 | 145 |  | Highly malignant or malignant patterns with delta or undetermined frequency < 7 days |  |  |  |
| **Keijzer,H.M** | 2021 | CPC1-2 vs CPC3-5 | cEEG | 3 | 0 | 17 | 30 | EEG Analyses | N/A | LX | JZH | Approved |
| **Westhall, E.** | 2016 | CPC1-2 vs CPC3-5 | rEEG | 38 | 0 | 38 | 27 | Table 3 | ≥1 highly malignant pattern | LX | JZH | Approved |
|  |  |  |  | 33 | 0 | 43 | 27 |  | Malignant periodic or rhythmic pattern |  |  |  |
| **Park J.S.** | 2023 | CPC1-2 vs CPC3-5 | rEEG | 30 | 0 | 18 | 28 | Table 3 | N/A | LJY | YYH | Approved |
| **Broman.N.J** | 2021 | CPC1-2 vs CPC3-5 | rEEG | 43 | 6 | 10 | 34 | Table 4 | N/A | JZH | LX | Approved |
| **Rossetti A. O.** | 2017 | CPC1-2 vs CPC3-5 | cEEG | 108 | 3 | 45 | 201 | Table 4 | Early EEG not  reactive | YYH | LJY | Approved |
|  |  |  |  | 123 | 48 | 30 | 156 |  | Early EEG not  continuous |  |  |  |
|  |  |  |  | 74 | 7 | 79 | 197 |  | Early EEG epileptiform |  |  |  |
|  |  |  |  | 105 | 3 | 48 | 201 |  | malignant  Early EEG highly |  |  |  |
|  |  |  |  | 96 | 1 | 57 | 203 |  | Late EEG not  reactive |  |  |  |
|  |  |  |  | 88 | 18 | 65 | 186 |  | Late EEG not continuous |  |  |  |
|  |  |  |  | 85 | 12 | 68 | 192 |  | Late EEG epileptiform |  |  |  |
|  |  |  |  | 96 | 3 | 57 | 201 |  | Late EEG highly malignant |  |  |  |
| **Kurt.Qing** | 2022 | CPC1-2 vs CPC3-5 | cEEG | 31 | 0 | 33 | 6 | Table4 | Day1 | JZH | LX | Approved |
|  |  |  |  | 30 | 0 | 34 | 6 |  | Day3 |  |  |  |
| **Peluso L.** | 2021 | CPC1-2 vs CPC3-5 | cEEG | 60 | 0 | 36 | 32 | Table 2 | HMp Day 1 | JZH | LX | Approved |
|  |  |  |  | 19 | 0 | 63 | 29 |  | HMp Day 2 |  |  |  |
|  |  |  |  | 76 | 9 | 20 | 23 |  | Unreactive EEG Day 1 |  |  |  |
|  |  |  |  | 48 | 3 | 34 | 26 |  | Unreactive EEG Day 2 |  |  |  |

(B)
